# Human Papillomavirus Types Distribution in Organised Cervical Cancer Screening in France

**DOI:** 10.1371/journal.pone.0079372

**Published:** 2013-11-14

**Authors:** Isabelle Heard, Laura Tondeur, Laurence Arowas, Michael Falguières, Marie-Christine Demazoin, Michel Favre

**Affiliations:** 1 French HPV Reference Laboratory, Institut Pasteur, Paris, France; 2 Université Pierre et Marie Curie University Paris 6; INSERM U943; Groupe Hospitalier Pitié-Salpêtrière, Paris, France; 3 Emerging Diseases Epidemiologic Expertise unit, Institut Pasteur, Paris, France; 4 Genetics, Papillomavirus and Human Cancer unit, Institut Pasteur, Paris, France; Georgetown University, United States of America

## Abstract

**Background:**

Knowledge of prevalence rates and distribution of human papillomavirus (HPV) genotypes prior high HPV vaccine coverage is necessary to assess its expected impact on HPV ecology and on cervical lesions and cancers.

**Methods:**

Residual specimens of cervical cytology (N = 6,538) were obtained from 16 sites participating in organised cervical cancer screening pilot programs throughout France, anonymised and tested for HPV DNA using the PapilloCheck® genotyping test. Samples were stratified according to age of women and cytological grades.

**Results:**

The age-standardised prevalence rates of HPV 16 and/or 18 (with or without other high-risk types) was 47.2% (95% Confidence Interval, CI: 42.4–52.1) in high-grade squamous intraepithelial lesions (HSILs), 20.2% in low-grade SIL (95% CI: 16.7–23.7) and 3.9% (95% CI: 2.8–5.1) in normal cytology. Overall HR HPV were detected in 13.7% (95%I CI: 11.7–15.6) of normal cytology. In women below 30 years of age, 64% of HSILs were associated with HPV16 and/or 18. In our study population, HPV16 was the most commonly detected type in all cervical grades with prevalence rates ranking from 3.0% in normal cytology to 50.9% in HSILs. HPV16 was also detected in 54% (27/50) of invasive cervical cancers including 5 adenocarcinomas.

**Conclusion:**

HPV16 was strongly associated with cervical precancer and cancer. The high prevalence rates of HPV16/18 infection among women below 30 years of age with HSILs suggests that the impact of vaccination would be primarily observed among young women.

## Introduction

Human papillomavirus (HPV) infection is frequent in young women and persistent infection may lead to cervical cancer. To date, more than 150 HPV genotypes have been characterized and about 40 of them infect the genital tract [1]. HPV are classified as low-risk and high-risk (HR) types according to their role in the development of cervical cancer. Epidemiological studies on the prevalence rates of genital HPV genotypes have shown that HPV16 and 18 induce about 70% of cervical cancers. Although the prevalence rates of HPV16 are roughly similar worldwide, some variations have been observed for other HR HPV [2].

Two vaccines, Gardasil (quadrivalent vaccine against HPV6, 11, 16 and 18, manufactured by Merck, Whitehouse Station NJ) and Cervarix (bivalent vaccine against HPV16 and 18, manufactured by Glaxo-SmithKline Biologicals, Rixensart, Belgium), have been developed for the prevention of cervical precancerous lesions and cancers related to vaccine types. In France, these vaccines have been recommended for girls aged 14–23 years since 2008.

Reliable baseline estimates of the prevalence of type specific cervical HPV infection in the general population are essential to assess the impact of vaccination on viral ecology and cervical lesions. In France, cervical cancer screening is reimbursed by the national health Insurance funds and recommendations are to perform a Pap test every 3 years for women aged 25–65. Regional organised screening pilot programs have been developed in Alsace since 2001 and were more recently introduced in various regions: Auvergne, Centre - Pays de Loire and Île-de-France. The increase in the population involved in organised screening was considered a good opportunity to obtain samples for HPV genotyping from the general population. On the whole, more than 6,000 cervical smears were collected and analysed at the French HPV Reference Laboratory for papillomaviruses (NRL HPV).

This study focuses on the distribution of HR HPV among women in France with normal and pathologic cytology findings and the age specific prevalence rates. Country-based type-specific HPV ecology provides baseline values against which the global impact of HPV vaccination might be assessed in the future. Furthermore, these data provide a critical reference measurement to assess unanticipated outcomes on virologic ecology and potential increases in non-vaccine HPV types.

## Materials and Methods

### Sample Collection

Liquid-based cytology (LBC) samples were collected prospectively from July 2009 through to November 2012 from women attending organised cervical screening in 16 pilot sites located in Alsace, Auvergne, Centre - Pays de Loire, Île-de-France and Vaucluse. Samples were collected and handled according to local protocols. After completion of cytology, residual LBC samples were anonymised and sent to the French HPV Reference Laboratory for HPV genotyping. Thinprep (Hologic), Easyfix (Labonord) and Surepath (Becton Dickinson) transport media were accepted. The following data were collected on the vial: date of birth, date of sampling, cytology result, and postal code.

For normal cytology, stratified sampling according to age and restricted to women aged 25 to 65 years (25–29, 30–39, 40–49, 50–65 years) was used to analyse HPV type distribution within each combination of age-band. For abnormal cytology, residual samples with abnormal cytology were collected irrespective of the age of the patient. For each region, the number of samples to be collected for each grade of abnormal smears was evaluated based on the rate and distribution of abnormal smears reported in the years preceding the study.

Cytological results were classified according to the Bethesda system 2001 [3]. Samples were classified as atypical squamous cell of undetermined significance (ASC-US), low-grade squamous intraepithelial lesions (LSILs), high-grade squamous intraepithelial lesions (HSILs) including atypical squamous cells – cannot exclude HSILs (ASC-H), adenocarcinoma in situ (AIS) and carcinoma. Cytology was evaluated by cytoscreeners and pathologists blinded to the outcomes of HPV testing.

### Isolation of DNA from Cervical Cells

Samples were divided in two identical aliquots (1 ml) and centrifuged at 14,000 rpm for 10 min. Pellets were suspended in 1 ml phosphate buffered saline, centrifuged (14,000 rpm for 10 min) and frozen. One pellet was stored at −20°C to account for potential problems in genotyping. Cells were lysed by an overnight incubation at 37°C in the presence of proteinase K (1.25 mg/ml) and DNA were extracted and purified with the NucleoSpin 96 tissue core kit (Macherey Nagel) according to the recommendations of the supplier. Two blank samples per plate (water without cervical cells) were processed as negative control to assess possible contamination.

### HPV Genotyping

HPV genotyping was carried out with the PapilloCheck® test system (Greiner BioOne GmbH, Frickenhausen, Germany). The assay identifies 13 HR HPV (HPV16, 18, 31, 33, 35, 39, 45, 51, 52, 56, 58, 59, and 68), five possibly oncogenic HPV (HPV53, 66, 70, 73, and 82) and six HPV considered as low risk types (HPV6, 11, 40, 42, 43, and 44/55). The assay uses multiplex PCR with fluorescent primers to amplify a DNA fragment of about 350 nucleotides within the E1 open reading frame of HPV genome. Amplification of an internal HPV template present in the PCR mastermix generated a signal on the PCR control spot on the chip. In addition, an internal PCR control which targets a region within the human ADAT-1 gene (adenosine deaminase tRNA specific 1) was used to assess the integrity of extracted DNA. The PCR reaction was performed with 5 microliters input cell DNA, the extraction blank samples and negative PCR controls (water) following the manufacturer’s recommendations. The amplification products were hybridized to specific HPV DNA probes fixed on the DNA chip. After washings, the chips were dried and automatically scanned and analyzed using the CheckScanner™ and the CheckReport ™ software developed by the manufacturer. A valid result was defined as one that passed all the internal controls.

### Data Analysis

HR HPV types were defined according to the 2009 International Agency Research on Cancer classification of types, which were at least “probably carcinogenic to humans”, that is, HPV 16, 18, 31, 33, 35, 39, 45, 51, 52, 56, 58, 59 and 68 [4]. Five groups of HPV infection were defined for the purpose of the analysis: (i) infection with any among the 13 HR HPV genotypes irrespective of low risk types, (ii) infection with HPV 16 and/or HPV 18 irrespective of the presence of any other type, (iii) infection with HPV 16 and/or 18 alone, (iv) infection with HR HPV genotypes other than HPV16 and/or 18 and (v) multiple infection with at least one HR type among positive samples with infection with any among the 13 HR HPV genotypes.

The prevalence rates of HPV infection for each disease grade was calculated with 95% confidence intervals (CIs) using sampling weights based on the distribution of the population (INSEE 2013). It was necessary to estimate prevalence rates for the screened population as we used stratified sampling based on age and cytology grade. The prevalence rates of HPV infection according to age was calculated with 95% CIs restricted to samples from women aged 25–65 years attending for cervical cancer screening program. Comparison of prevalence rates between age groups and region were based on the chi^2^ test. Data were analysed with STATA Software version 12.0 (STATA Corporation, College station, Texas, USA).

### Ethical Considerations

This study was designed as a quality development study, utilizing only residual material that would otherwise have been discarded. According to French regulations of biomedical research, an ethical approval is not necessary for such studies. The study was given favourable ethical opinion by the Comité de Recherche Clinique at the Institut Pasteur (Avis n° 2009–51).

## Results

**Figure 1:**
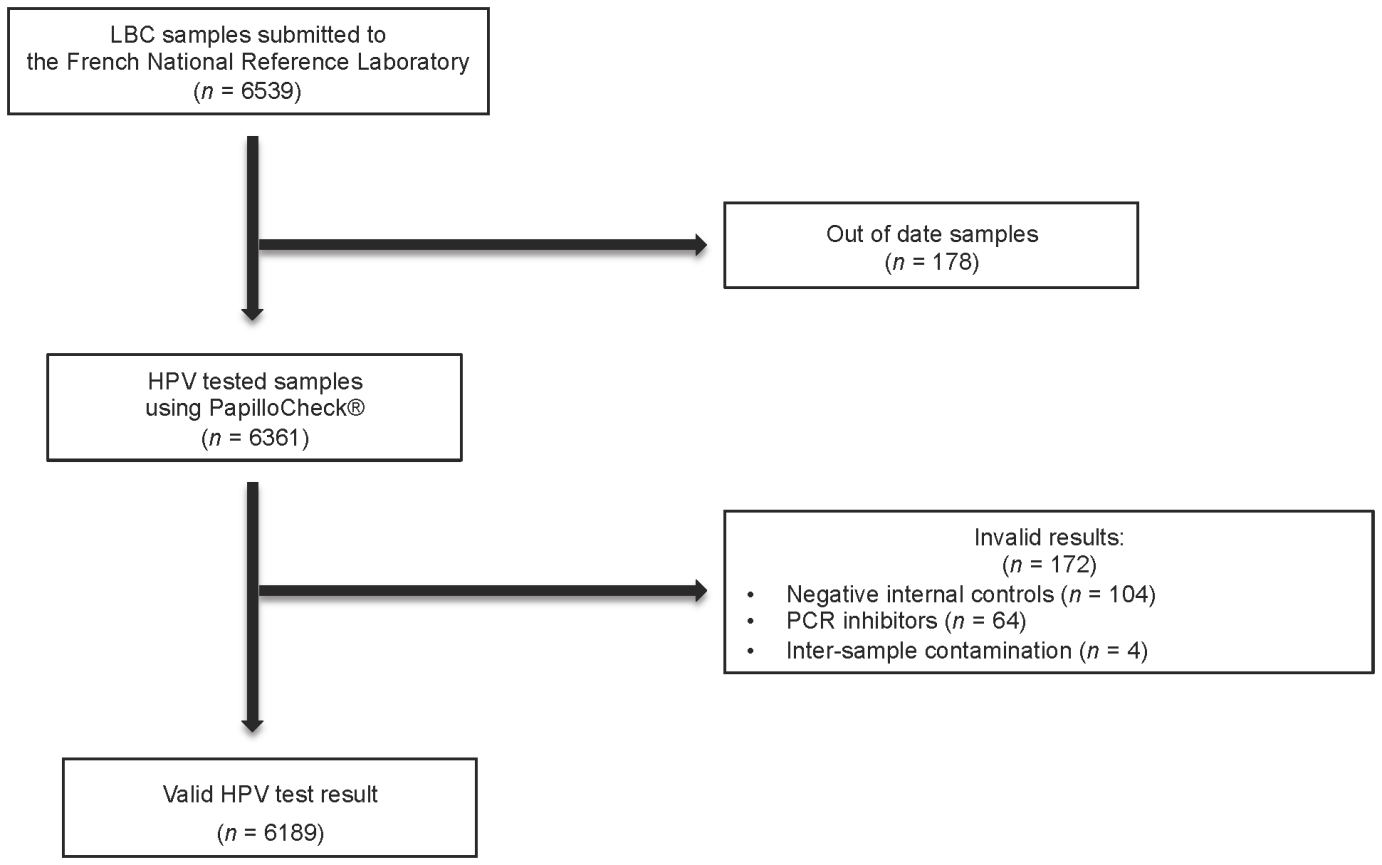
Sample Submission and Human Papilloma Virus (HPV) Testing Algorithm for Residual Liquid Based Cytology (LBC) Samples.

A total of 6,539 samples were received at the National Reference Laboratory. For 178 samples (2.7%), the duration between collection and arrival at the NRL exceeded 4 weeks of storage recommended by the manufacturer before genotyping. These out of date samples were excluded of the study. Invalid genotyping results were obtained for 172 (2.7%) of the 6,361 remaining samples. They corresponded to 104 (1.6%) samples with negative internal controls, and 64 (1.0%) samples with PCR inhibitors. In addition, contamination could not be excluded for four DNA preparations (0.006%) since mixtures occurred during processing of samples (Figure 1).

At total of 6,189 samples were found adequate for genotyping. Table 1 presents the geographic location of participants and the cytologic grades of samples. The mean age of the study population was 39.4 years. A total of 5367 women (86.7%) belonged to the target age group of 25–65 years, for whom screening is recommended in France, whereas 588 women (9.5%) were younger than 25 years and 234 (3.8%) were older than 65 years.

**Table 1:** Geographic location of participants and cytology grade of samples.

	n = 6189	Median Age (interquartile range)
*Region:*		
Alsace	1563	36.3
		(28.3–45.9)
Auvergne	1363	39
		(29.3–48.8)
Centre - Pays de Loire	1610	38.9
		(30.3–49.9)
Ile de France	1535	35.8
		(28.2–45.6)
Vaucluse	118	36.2
		(25.9–43.6)
*Cytology grade:*		
Normal	3023	40.1
		(30.3–51.1)
ASC-US	1070	35.2
		(26.9–44.1)
LSIL	1179	32.1
		(26.1–42.6)
HSIL	867	37.1
		(30.6–45.7)
Cancer	50	48.2
		(37.2–61.7)

HR HPV, High Risk HPV; ASC-US, Atypical Squamous Cells of Undetermined significance; LSIL, Low grade Squamous Intra-epithelial Lesion; HSIL, High grade Squamous Intra-epithelial Lesion.

The age-standardised prevalence rates of each of the five defined cytology categories of HR HPV, ranging from normal to HSILs, are shown in Table 2. The prevalence rates of HR HPV infection, HPV 16 and/or 18 infection irrespective of other HPV types and of HPV 16 and/or 18 alone increased significantly with increasing severity of cytology grade from 13.7 (95% CI: 11.7–15.6), 3.9 (95% CI: 2.8–5.1) and 2.7 (95% CI: 1.7–3.6) respectively among women with normal cytology to 84.4 (95% CI: 80.2–88.6), 47.2 (95% CI: 42.4–52.1) and 27.2 (95% CI: 22.9–31.5) in HSILs. On the opposite, HR HPV infection other than HPV16/18 and multiple infections did not increase with cytology grades and were higher in LSILs than in HSILs (48.7% and 37.1%, respectively for HR HPV infection other than HPV16/18 and 49.1 and 39.9 respectively for multiple infections).

**Table 2:** Age standardized infection rate by cytology grade.

HPV status	Cytology								P =
	Normal		ASC-US		LSIL		HSIL		
	n = 3023		n = 1070		n = 1179		n = 867		
	%	95% CI	%	95% CI	%	95% CI	%	95% CI	
HR HPV	13.7	11.7–15.6	48.3	41.4–55.3	68.9	64.4–73.5	84.4	80.2–88.6	<0.001
HPV 16 and/or 18 irrespectiveof other HPV type	3.9	2.8–5.1	19.2	12.9–25.5	20.2	16.7–23.7	47.2	42.4–52.1	<0.001
HPV 16 and/or 18 alone	2.7	1.7–3.6	6.4	2.7–10.1	5.9	4.0–7.8	27.2	22.9–31.5	<0.001
HR HPV other than HPV16 and/or 18	9.7	8.0–11.4	29.1	23.2–35.1	48.7	43.9–53.5	37.1	32.0–42.2	<0.001
Multiple infections with at least oneHR HPV among HPV positive samples	23.0	18.1–28.0	47.5	38.8–56.3	49.1	44.2–54.0	39.9	34.3–45.4	<0.001

HR HPV, High Risk HPV; ASC-US, Atypical Squamous Cells of Undetermined Significance; LSIL, Low grade Squamous Intra-epithelial Lesion; HSIL, High grade Squamous Intra-epithelial Lesion; CI, Confidence Interval.

The prevalence rates of the five defined categories of HR HPV by age-group among the 5326 women participating to the study and belonging to the 25–65 target age group for cervical cancer screening are shown in Table 3. There was a marked decline in the prevalence rates of HR HPV and of HPV16 and/or 18 irrespective of other HPV types with age, from 46.0% (95% CI: 43.6–48.3), and 20.1% (95% CI: 18.2–22.1), respectively in the 30–39-year age group to 26.4% (95% CI: 23.8–29.3) and 8.6% (95% CI: 7.0–10.5) in women aged 50–64 years. (p<0.001). Rates of infection with HR HPV other than HPV16 and/or 18 and of multiple infections decreased similarly significantly with increasing age (p<0.001).

**Table 3:** Prevalence of HR HPV by age group among the 5326 women eligible for routine screening (age 25–65).

HPV status	Age group (years)								P =
	25–29		30–39		40–49		50–64		
	n = 1204		n = 1701		n = 1400		n = 1021		
	Infectionrate (%)	95% CI	Infectionrate (%)	95% CI	Infectionrate (%)	95% CI	Infectionrate (%)	95% CI	
HR HPV	44.5	41.7–47.4	46.0	43.6–48.4	36.4	33.9–39.0	26.4	23.8–29.3	<0.001
HPV 16 and/or 18 irrespective ofother HPV type	19.7	17.5–22.0	20.1	18.2–22.1	15.5	13.6–17.5	8.6	7.0–10.5	<0.001
HPV 16 and/or 18 alone	7.7	6.3–9.4	10.6	9.2–12.1	8.1	6.7–9.6	4.7	3.5–6.2	<0.001
HR HPV other than HPV16 and/or 18	24.8	22.4–27.4	25.9	23.8–28.0	20.9	18.8–23.2	17.8	15.5–20.3	<0.001
Multiple infections with at least one HR HPV among HPV positive samples	48.3	44.3–52.2	40.4	37.2–43.6	37.6	33.8–41.5	34.5	29.7–39.6	<0.001

HR HPV, High Risk HPV; CI, Confidence Interval.

**Figure 2:**
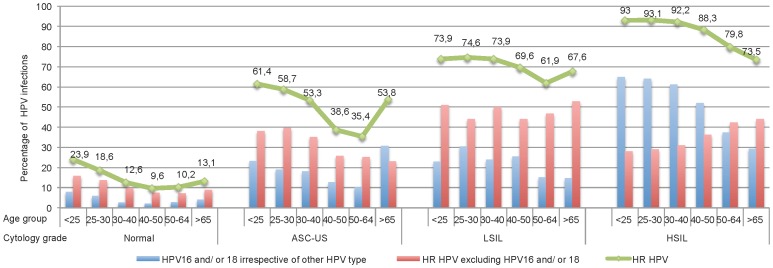
Age specific prevalence of HPV infection in different cytology groups. Prevalence of HPV infection High Risk (HR) but not 16 and/or 18 and HPV16 and/or 18 without or with another HPV type by cervical grade and age group. ASC-US, Atypical Squamous Cells of Undetermined Significance; LSIL, Low Grade Squamous Intra-epithelial Lesion; HSIL, High Grade Squamous Intra-epithelial Lesion.

Figure 2 shows the prevalence rates of HR HPV infection, infection with HR HPVs other than HPV16 and 18 and infection with HPV 16 and/or 18 irrespective of other HPV types by disease-grade in each age-band. Regardless of the grade of cytology, the overall rate of HR HPV infection decreased with age. In ASC-US, the largest drop was observed after the age of 40 (57.2% in women below 40 years versus 38.1% in older women, p<0.001). For normal Pap tests, ASC-US and LSILs, the rate of infection with HR HPVs other than HPV16 and 18 was always higher than the rate of infection with HPV 16 and/or 18 irrespective of other HPV types, whatever the age. By contrast, in HSILs the rate of infection with HPV 16 and/or 18 irrespective of other HPV types exceeded the rate of infection with HR HPVs excluding HPV16 and 18 among women aged less than 50 years, while the opposite was true among older women. In normal smears, ASC-US and LSILs, a slight increase in the rate of HR HPV infection was observed in women over 65 years compared to women aged 50–64.

Prevalence rates of HPV16 and/or 18 and of HR HPV other than HPV16 and/or 18 in HSILs did not vary significantly by region (Data not shown).

Overall, multiple infection with at least one HR type was detected in 40.0% of all HPV-positive samples (95% CI: 37.3–42.8). Nearly half of HR-HPV infections were multiple in low-grade lesions (49.1%, 95% CI: 44.2–54.0, in LSILs and 47.5%, 95% CI: 38.8–56.3, in ASC-US) against 39.9% (95% CI: 34.3–45.4) in HSILs and only 23.0% (95% CI: 18.1–28.0) in normal smears (Table 2). Among women eligible for routine screening and infected with HR HPV, the age-specific prevalence rates of multiple infections decreased with increasing age from 48.3% (95% CI: 44.3–52.2) among women aged 25–29 to 34.5% (95% CI: 29.7–39.6) among women in the 50–64 age group (p<0.001) (Table 3).

Prevalence rates for each of the 13 HR HPV types, for the 5 possibly oncogenic types and for LR HPV6 and 11 detected with PapilloCheck® are shown in Table 4 both overall and by cervical disease grade. Overall, HPV16 was detected in 15.2% of the study population and was the most commonly detected type in all cervical grades. HPV18 ranked at the 12^th^ place (2.4%). HPV16 and/or 18 were detected in 3.5% of samples without cytologic abnormalities, 17.1% of ASC-US, 24.1% of LSILs and 55.6% of HSILs. The second more prevalent genotype detected was HPV51 in women with normal cytology, HPV53 in ASC-US and LSILs and HPV31 in HSILs. The HPV6 and HPV11genotypes were only detected in 1.9% and 0.4% of samples, respectively.

**Table 4:** Human papillomavirus types by cytology grades (n = 6139).

HPV type	Cytology								TOTAL	
	Normal (n = )		ASC-US		LSIL		HSIL			
	N+	% (N+/tot)	N+	% (N+/tot)	N+	% (N+/tot)	N+	% (N+/tot)	N+	% (N+/tot)
16	91	3.0	157	14.7	246	20.9	441	50.9	935	15.2
18	16	0.5	30	2.8	49	4.2	51	5.9	146	2.4
16/18	106	3.5	183	17.1	284	24.1	482	55.6	1055	17.2
31	37	1.2	86	8.0	133	11.3	106	12.2	362	5.9
33	14	0.5	22	2.1	34	2.9	58	6.7	128	2.1
35	7	0.2	18	1.7	31	2.6	25	2.9	81	1.3
39	40	1.3	66	6.2	97	8.2	46	5.3	249	4.1
45	21	0.7	33	3.1	34	2.9	39	4.5	127	2.1
51	86	2.8	83	7.8	188	15.9	83	9.6	440	7.2
52	34	1.1	68	6.4	89	7.6	66	7.6	257	4.2
53	50	1.6	98	9.2	200	16.9	51	5.9	399	6.5
56	61	2.0	83	7.8	191	16.2	56	6.5	391	6.4
58	18	0.6	53	4.9	66	5.6	51	5.9	188	3.1
59	19	0.6	38	3.6	67	5.7	23	2.6	147	2.4
66	37	1.2	54	5.1	179	15.2	39	4.5	309	5.0
68	32	1.1	45	4.2	78	6.6	40	4.6	195	3.2
70	16	0.5	25	2.3	34	2.9	28	3.2	103	1.7
73	9	0.3	29	2.7	42	3.6	14	1.6	94	1.5
82	8	0.3	24	2.2	42	3.6	25	2.9	99	1.6
6	11	0.4	39	3.6	49	4.2	15	1.7	114	1.9
11	1	0.03	10	0.9	7	0.6	6	0.7	24	0.4

HPV types were detected with the PapilloCheck® technique. N+: Number of positive samples. ASC-US, Atypical Squamous Cells of Undetermined Significance; LSIL, Low grade Squamous Intra-epithelial Lesion; HSIL, High grade Squamous Intra-epithelial Lesion; tot, total.

### Cervical Cancer

Fifty cases of cytologic diagnoses of cervical cancer were analysed. The mean age of patients was 50.4 years. HPV could not be detected in 1 of the 5 adenocarcinomas and in 3 of the 45 squamous cell carcinomas. HPV 16 was detected in 27 cases (54%) and was associated with other genotypes in 9 of the 27 cases. HPV53 and HPV66 were detected in 6 cases each, and were the second more frequently detected types. HPV 18 was detected in neither the adenocarcinomas nor the squamous cell carcinomas.

## Discussion

The present study is the first large population study of women in France to have undergone HPV testing and genotyping in the context of an organised cervical cancer program. Although from a limited geographic area, women enrolled in the study are representative of women across the cervical cancer screening program. Seventy five of them were located in districts or regions initiating a pilot program while the others were living in Alsace where screening has been organised for several years.

We observed that 13.7% of normal Pap tests were positive for HR HPV types. Rates above 10% were also observed in studies performed in UK and Belgium and using PCR-based methods [5] [6]. A lower pooled prevalence rate of 8.4% of HPV infection in normal cytology in Western Europe was found by de San José in a meta-analysis using prevalence data obtained with PCR assays and Hybrid Capture2 (Qiagen) [7]. Part of the differences observed between studies may be attributable to the use of different HPV tests with heterogeneity in sensitivity and specificity [8].

We observed similar prevalence rates of HR HPV infection in LSILs and HSILs to those reported in other European studies and derived from meta-analyses although part of ASC-H samples might not have been true high-grade lesions [9] [10]. Indeed, the protocol did not allow us to have access to the histological diagnosis of lesions.

The overall prevalence rates of HR HPV infection and of HPV16 and/or 18 decreased with age among women below 64 as observed in several studies [11]. A slight increase was detected among older women with normal cytology and low-grade lesions. A similar U-shaped curve has been observed in other studies and in Latin American countries [12] [13]. Several hypotheses have been made about this second peak in older women including changes in sexual behaviour in middle age women and/or different lifetime number of sexual partners [7] [14]. Women in our study were not interviewed about their sexual behaviour. We were therefore unable to attribute this increase to the acquisition of new infections or reactivation of a latent persistent infection that becomes detectable in the menopausal context [15]. If confirmed, this rise, as well as a rate of 4.1% of infection with HPV16 and/or 18 among women aged over 65 years with normal cytology, could lead to reconsider the age at ending screening for cervical cancer.

We observed age-dependent prevalence rates of HR HPV in ASC-US. In France, current guidelines for the management of ASC-US recommend immediate colposcopy or an option of triage regardless of patient’s age (ANAES 2002). Pooled HR-HPV DNA testing and referring for immediate colposcopy only those who are also HR-HPV positive is an alternative, the second being a repeat Pap testing within 6 months and referring for immediate colposcopy only those with abnormal cytology. The large drop observed in ASC-US after the age of 40 years suggests that 40 might be a suitable age cut-off point for HR-HPV triage in ASC-US. Management of ASC-US using HR-HPV triage for women aged 40 years or older might be more relevant since only 38% of them will be HR-HPV positive, whereas the HR-HPV negative group comprises two thirds of women who will not need colposcopy and potential overtreatment.

Another finding of our study was that in HSILs, there was a shift in the predominant HPV types group with increasing age. The prevalence rates of infection with HPV16 and/or 18 irrespectively of other HR types decreased with age, while the prevalence rates of infection with HR HPV excluding HPV16/18 increased. This suggests that the impact of vaccination would be primarily observed in women below 30 years since the proportion of lesions associated with HPV16/18 types reached 64% is in this age group whereas infection with other HPV types excluding HPV16 and 18 were detected in less than 30%. Similar higher rates of infection with HPV16 and 18 were observed in CIN2–3 among younger women in Denmark [16]. Moreover, preliminary results of the impact of vaccination in HSILs have been observed in young women in a recently published Australian study [17].

Overall, the four most prevalent types in women without cytologic abnormalities were HPV16 (3.0%), 51 (2.8%), 56 (2.0%), and 53 (1.6%), whereas in HSILs the most prevalent types were HPV16 (50.9%), 31 (12.2%), 51 (7.2%) and 33 (6.7%). Epidemiologic distribution was quite different in a retrospective study performed in France with HPV16 being detected in 62% of CIN2/3 [18]. This difference could be explained by geographical variation in the screened population. A recombinant nine-valent vaccine is currently under development and will contain the following types: HPV6/11/16/18/31/33/45/52/58. Such a vaccine may not protect against the types 51, 56 and 53, respectively third, sixth and seventh more frequently detected in HSILs in our country [19].

Because cytology and virology results were available for all samples, we were able to assess the overall effect of vaccination on cervical cytology. The currently available prophylactic HPV vaccines provide protection against HPV16 and 18. In the event of a high coverage rate, one can assume that HPV16 and 18 would dramatically decrease or be eliminated together with all associated lesions. Furthermore, it has been reported that HPV16 and 18 vaccines could protect against lesions associated with closely related HPVs such as HPV31 and HPV45. However, lesions associated with other unrelated HR genotypes would persist at the same level. The percentages of cases of ASC-US and LSILs with HPV16 and/or 18 only were 6.4 and 5.9, respectively. Rates of HPV16 and/or 18 irrespective of other HR type reached 19.2% and 20.2% for ASC-US and LSILs, respectively. Depending on the attributable fraction of cases related to HPV16/18 in case of multiple infections including HPV16/18, the percentage of ASC-US and LSILs would fall between 6 and 20% in case of high HPV16/18 immunization of the population. The large majority of low grade lesions will thus still occur in vaccinated women. This is consistent with data from clinical trials of both prophylactic vaccines, which show greater efficacy in preventing high grade than low-grade lesions [20]. In fact, our data suggest a much higher reduction of high grade lesions ranking between 27 and 47%.

Our study has some limitations. First, since there is no national organised cervical cancer screening program in France, we were not able to collect samples representative of the country. Nevertheless, we were able to obtain specimens from different geographic areas and levels of urbanization including Paris agglomeration, residential and rural areas. Second, this is a cross-sectional study and correlation between HPV types and lesions should be interpreted with caution, especially since there is no histological confirmation of the lesions. Furthermore, the high rate of multiple infections makes it difficult to attribute a lesion to a specific type.

In conclusion, this is the first study to describe comprehensively type-specific prevalence rates across France in all cervical grades including normal. These results will help to estimate the proportion of disease potentially preventable with the current and second-generation vaccines. They will also contribute to the evaluation of the impact of HPV vaccination on vaccine type prevalence and possible cross-protection or type replacement.

## References

[1] Bernard HU, Burk RD, Chen Z, van Doorslaer K, zur Hausen H, et al (2010) Classification of papillomaviruses (PVs) based on 189 PV types and proposal of taxonomic amendments. Virology 401: 70–79. 20206957 10.1016/j.virol.2010.02.002 PMC3400342

[2] Clifford GM, Smith JS, Plummer M, Munoz N, Franceschi S (2003) Human papillomavirus types in invasive cervical cancer worldwide: a meta-analysis. Br J Cancer 88: 63–73. 12556961 10.1038/sj.bjc.6600688 PMC2376782

[3] Solomon D, Davey D, Kurman R, Moriarty A, O'Connor D, et al (2002) The 2001 Bethesda System: terminology for reporting results of cervical cytology. JAMA 287: 2114–2119. 11966386 10.1001/jama.287.16.2114

[4] Bouvard V, Baan R, Straif K, Grosse Y, Secretan B, et al (2009) A review of human carcinogens–Part B: biological agents. Lancet Oncol 10: 321–322. 19350698 10.1016/s1470-2045(09)70096-8

[5] Arbyn M, Benoy I, Simoens C, Bogers J, Beutels P, et al (2009) Prevaccination distribution of human papillomavirus types in women attending at cervical cancer screening in Belgium. Cancer Epidemiol Biomarkers Prev 18: 321–330. 19124515 10.1158/1055-9965.EPI-08-0510

[6] Howell-Jones R, Bailey A, Beddows S, Sargent A, de Silva N, et al (2010) Multi-site study of HPV type-specific prevalence in women with cervical cancer, intraepithelial neoplasia and normal cytology, in England. Br J Cancer 103: 209–216. 20628396 10.1038/sj.bjc.6605747 PMC2906740

[7] de Sanjose S, Diaz M, Castellsague X, Clifford G, Bruni L, et al (2007) Worldwide prevalence and genotype distribution of cervical human papillomavirus DNA in women with normal cytology: a meta-analysis. Lancet Infect Dis 7: 453–459. 17597569 10.1016/S1473-3099(07)70158-5

[8] Bruni L, Diaz M, Castellsague X, Ferrer E, Bosch FX, et al (2010) Cervical human papillomavirus prevalence in 5 continents: meta-analysis of 1 million women with normal cytological findings. J Infect Dis 202: 1789–1799. 21067372 10.1086/657321

[9] Clifford GM, Rana RK, Franceschi S, Smith JS, Gough G, et al (2005) Human papillomavirus genotype distribution in low-grade cervical lesions: comparison by geographic region and with cervical cancer. Cancer Epidemiol Biomarkers Prev 14: 1157–1164. 15894666 10.1158/1055-9965.EPI-04-0812

[10] Smith JS, Lindsay L, Hoots B, Keys J, Franceschi S, et al (2007) Human papillomavirus type distribution in invasive cervical cancer and high-grade cervical lesions: a meta-analysis update. Int J Cancer 121: 621–632. 17405118 10.1002/ijc.22527

[11] Sargent A, Bailey A, Almonte M, Turner A, Thomson C, et al (2008) Prevalence of type-specific HPV infection by age and grade of cervical cytology: data from the ARTISTIC trial. Br J Cancer 98: 1704–1709. 18392052 10.1038/sj.bjc.6604324 PMC2391119

[12] Franceschi S, Herrero R, Clifford GM, Snijders PJ, Arslan A, et al (2006) Variations in the age-specific curves of human papillomavirus prevalence in women worldwide. Int J Cancer 119: 2677–2684. 16991121 10.1002/ijc.22241

[13] Smith JS, Melendy A, Rana RK, Pimenta JM (2008) Age-specific prevalence of infection with human papillomavirus in females: a global review. J Adolesc Health 43: S5–25, S25 e21–41. 10.1016/j.jadohealth.2008.07.009 18809145

[14] Gravitt PE, Rositch AF, Silver MI, Marks MA, Chang K, et al (2013) A cohort effect of the sexual revolution may be masking an increase in human papillomavirus detection at menopause in the United States. J Infect Dis 207: 272–280. 23242540 10.1093/infdis/jis660 PMC3532829

[15] Althoff KN, Paul P, Burke AE, Viscidi R, Sangaramoorthy M, et al (2009) Correlates of cervicovaginal human papillomavirus detection in perimenopausal women. J Womens Health (Larchmt) 18: 1341–1346. 19702476 10.1089/jwh.2008.1223 PMC2825723

[16] Baandrup L, Munk C, Andersen KK, Junge J, Iftner T, et al (2012) HPV16 is associated with younger age in women with cervical intraepithelial neoplasia grade 2 and 3. Gynecol Oncol 124: 281–285. 22036987 10.1016/j.ygyno.2011.10.020

[17] Brotherton JM, Fridman M, May CL, Chappell G, Saville AM, et al (2011) Early effect of the HPV vaccination programme on cervical abnormalities in Victoria, Australia: an ecological study. Lancet 377: 2085–2092. 21684381 10.1016/S0140-6736(11)60551-5

[18] Pretet JL, Jacquard AC, Saunier M, Clavel C, Dachez R, et al (2008) Human papillomavirus genotype distribution in low-grade squamous intraepithelial lesions in France and comparison with CIN2/3 and invasive cervical cancer: the EDiTH III study. Gynecol Oncol 110: 179–184. 18514800 10.1016/j.ygyno.2008.04.012

[19] Serrano B, Alemany L, Tous S, Bruni L, Clifford GM, et al (2012) Potential impact of a nine-valent vaccine in human papillomavirus related cervical disease. Infect Agent Cancer 7: 38. 23273245 10.1186/1750-9378-7-38 PMC3554470

[20] Schiller JT, Castellsague X, Garland SM (2012) A review of clinical trials of human papillomavirus prophylactic vaccines. Vaccine 30 Suppl 5F123–138. 23199956 10.1016/j.vaccine.2012.04.108 PMC4636904

